# A novel target for analgesic substances: physiological role of Na,K-ATPase as the signal transducer

**DOI:** 10.3389/fnmol.2025.1717676

**Published:** 2025-11-26

**Authors:** Valentina A. Penniyaynen, Dmitriy M. Samosvat, Vera B. Plakhova, Svetlana A. Podzorova, Anna V. Berintseva, Irina P. Butkevich, Viktor A. Mikhailenko, Georgy G. Zegrya, Ke Ma, Ilya V. Dukhovlinov, Boris V. Krylov, Ilya V. Rogachevskii

**Affiliations:** 1 Laboratory of Physiology of Excitable Membranes, Pavlov Institute of Physiology, Russian Academy of Sciences, Saint Petersburg, Russia; 2 Ioffe Institute, Russian Academy of Sciences, Saint Petersburg, Russia; 3 Department of Pain Management, Xinhua Hospital, Shanghai Jiao Tong University School of Medicine, Shanghai, China; 4 ATG Service Gene LLC, Saint Petersburg, Russia

**Keywords:** ouabain–Ca^2+^ chelate complex, Na, K-ATPase signaling function, Nav1.8 channel, formalin test, organotypic tissue culture method, docking, nociception, analgesics

## Abstract

A potential analgesic medicinal substance has been discovered, the ouabain–Ca^2+^ chelate complex (EO). As we have found, the specific EO binding to the Na,K-ATPase (NKA) in nanomolar concentrations triggers several signaling cascades in the nociceptive neuron, two of which have been discussed elsewhere. The docking results indicate that the molecular basis for the specificity of EO–NKA binding is the formation of two intermolecular ionic bonds between the chelated Ca^2+^ cation and two NKA carboxylate anion, Glu116 and Glu117. The third downstream EO-triggered NKA/Src/PKA/p38 MAPK/NF-κB signaling pathway, likely, controls the GAP43 gene expression, which results in this case in the neurite-inhibiting effect at the tissue level. The strong EO analgesic effect at both the spinal and supraspinal levels has been demonstrated in the formalin test. EO is a promising candidate for the role of a novel and safe analgesic, which might be particularly effective for the treatment of the tumor-associated pain syndromes due to its possible cytostatic function.

## 1 Introduction

Ouabain is a naturally occurring cardiotonic steroid first extracted from the *Strophanthus gratus* plant (Arnaud, 1888). The suggested evolutionary function of plant-derived ouabain is the protection from herbivorous animals (El-Mallakh et al., 2021). The ouabain level in plants is therefore superphysiological for mammals and can be toxic to them due to the inhibition of the Na,K-ATPase (NKA) pumping function at micromolar concentrations (Glynn, 1957). However, this effect has found the medicinal use more than two centuries ago for the treatment of various heart dysfunctions (Page, 1964). Rather recently, it has become clear that ouabain is also endogenously produced in the mammalian adrenal gland and hypothalamus in nanomolar concentrations (Blaustein, 2018; El-Masri et al., 2002; Hamlyn et al., 1991; Kawamura et al., 1999; Lichtstein et al., 1998; Takahashi et al., 1994; Tegin et al., 2021). Ouabain has been demonstrated to modulate the NKA non-pumping (signaling) function which activates an extensive network of signaling cascades that control various functions in different cell types (Blaustein and Hamlyn, 2020; Blaustein and Hamlyn, 2024).

Our findings unambiguously indicate that the ouabain–Ca^2+^ chelate complex (EO) exhibits a strong antinociceptive effect associated with the modulation of the Na_*V*_1.8 channel functional activity in the nociceptive neuron membrane (Plakhova et al., 2020). According to the current data, these channels are the molecular markers of nociceptive neurons (Bennett et al., 2019). Previously, we have shown that the Na_*V*_1.8 channel is the effector unit of the tangential membrane signaling cascade triggered by EO binding to NKA, which serves both as the EO receptor and the signal transducer (Krylov et al., 2017). EO also triggers the downstream NKA/Src/PKA/p38 MAPK signaling to the neuron genome, which decreases the density of the Na_*V*_1.8 channels in the nociceptive neuron membrane via modulation of the SCN10A gene that controls the Na_*V*_1.8 channel expression (Plakhova et al., 2020). At the same time, there is an additional EO-triggered downstream NKA/Src/PKA/p38 MAPK pathway which regulates the inhibition of dorsal root ganglia (DRG) neurite growth (Plakhova et al., 2020). It has to be stressed that the statistically significant neurite-inhibiting effect of EO necessarily accompanies its dual antinociceptive effect. Therefore, a more detailed investigation of this additional pathway is required to further elucidate the physiological consequences of its activation at the cellular level.

**FIGURE 1:**
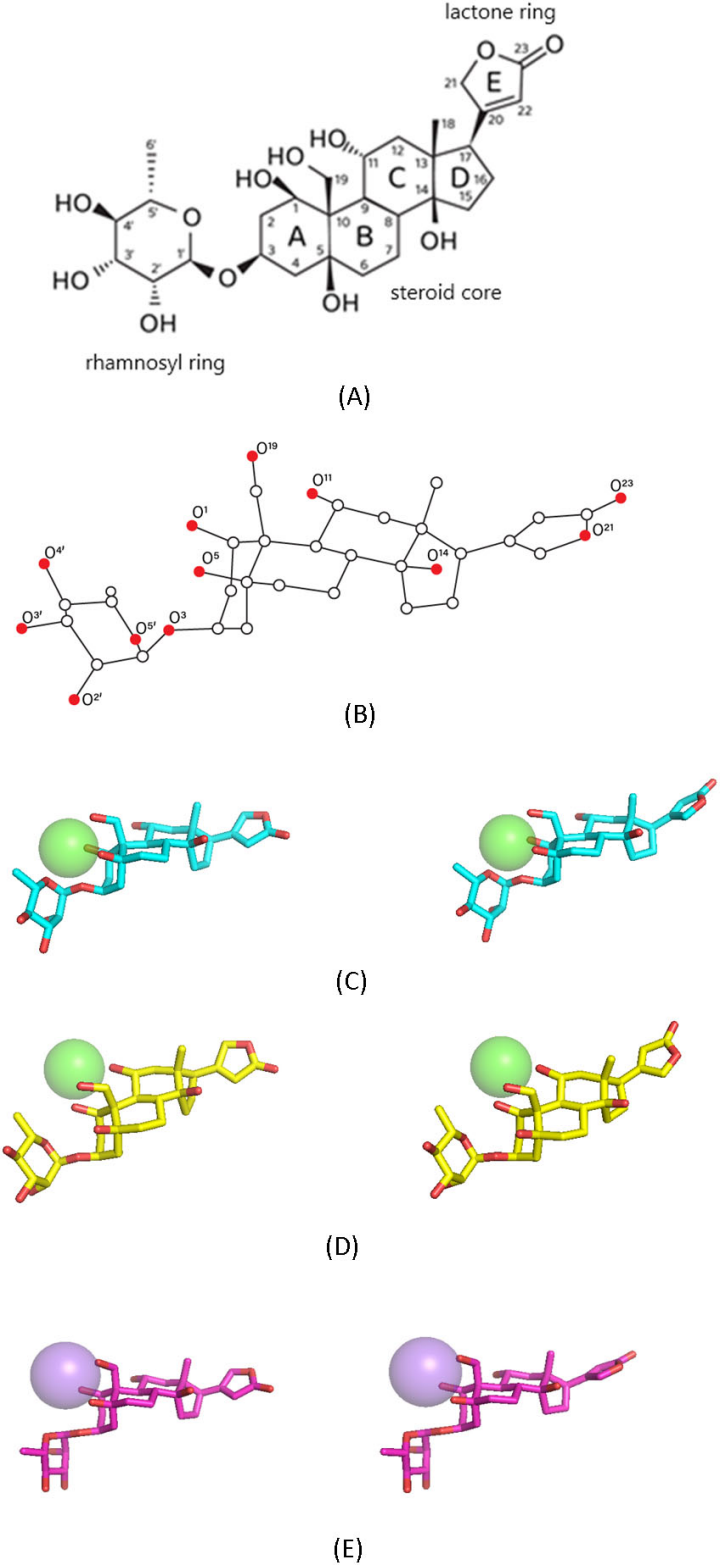
Structural data on ouabain and ligand conformations used for docking with NKA. **(A)** Chemical structure of ouabain, designation of the rings, and numbering of carbon atoms. **(B)** Three-dimensional structure of ouabain and numbering of oxygen atoms. Carbon, white spheres; oxygen, red spheres. **(C)** EO5. Carbon, cyan; oxygen, red; calcium, green sphere. The calcium cation is chelated by five (O^1^, O^3^, O^5^, O^19^, O^5^′) ouabain oxygens. **(D)** EO3. Carbon, yellow; oxygen, red; calcium, green sphere. The calcium cation is chelated by three (O^1^, O^11^, O^19^) ouabain oxygens. **(E)** OUA-Na. Carbon, magenta; oxygen, red; sodium, violet sphere. The sodium cation is chelated by three (O^1^, O^5^, O^19^) ouabain oxygens. The difference between the left and right conformations is the E ring orientation relative to the steroid core (rings A–D). Hydrogen atoms are not shown.

In physiological conditions, the downstream EO signaling in the nociceptive neuron is blocked by the selective calcium chelator EGTA (Plakhova et al., 2020), which supports the idea that EO is rather the ouabain–Ca^2+^ chelate complex than the free ouabain molecule (Figures 1A,B). The ouabain–Ca^2+^ chelate complex is an individual molecule that is structurally very close to ouabain and contains a divalent Ca^2+^ cation coordinated by several oxygen atoms (Figures 1C,D). However, it is very difficult to structurally differentiate EO from the free ouabain molecule (OUA) using experimental methods. Therefore, only theoretical calculations methods can provide a reliable insight into the molecular mechanism of EO-NKA binding.

Docking of OUA with two NKA models has demonstrated that it forms 10–12 intermolecular hydrogen bonds, all of which are required for the energetically optimal ouabain binding (Rogachevskii et al., 2023). This hydrogen bond network involves almost all oxygen atoms that are relatively evenly distributed over the entire ouabain molecule, and rather strictly determines the ligand position within its unique NKA binding site. Because both OUA and EO bind to the same NKA site, the chelated Ca^2+^ cation is expected to be involved in strong energetically favorable interactions upon EO binding to NKA. Most logically, the cation electrostatically interacts with the anionic NKA residues. These intermolecular ionic bonds are evidently absent upon the OUA binding to NKA, and their formation should induce a conformational change in the entire NKA molecule and noticeably contribute to the EO-NKA binding energy, thus allowing for EO to modulate the NKA signaling function as opposed to OUA.

Quite clearly, investigation of NKA signaling can help create new medicinal substances (Xie and Xie, 2005), and EO is thus a promising candidate for the role of a potent analgesic. Due to its pronounced neurite-inhibiting effect, EO may be particularly effective for the treatment of cancer-related pain syndrome accompanied with uncontrolled neural tissue growth. Our results indicate that ouabain, being converted into EO at nanomolar concentrations, might play a novel and direct physiological role *in vivo* as an analgesic agent.

It must be the delicate mechanism of Ca^2+^ chelation that is mandatory for the implementation of the specific EO-triggered activation of the signaling rather than the pumping function of NKA in the nociceptive neuron. To collect evidence in support of this idea, a number of theoretical and experimental methods have been applied. Four stable EO conformations (Figures 1C,D) and two stable conformations of the ouabain-Na^+^ chelate complex (OUA-Na) (Figure 1E), used as a reference structure with the monovalent Na^+^ cation instead of the divalent Ca^2+^, were docked with two NKA models to elucidate the molecular basis for the EO effect. The analgesic effect of ouabain preincubated with either Ca^2+^ or Na^+^ was studied *in vivo* using the formalin test.

Finally, the combined application of organotypic tissue culture, confocal laser microscopy, and immunofluorescence image analysis techniques made it possible to identify another consecutive unit in the EO-triggered NKA/Src/PKA/p38 MAPK downstream signaling pathway, the NF-κB transcription factor. We hypothesize that the NF-κB activation modulates the expression of two genes at the same time: the SCN10A gene that controls the density of the Na_*V*_1.8 channels in the nociceptive neuron membrane (Plakhova et al., 2020), and the GAP43 gene that controls the inhibition of DRG neurite growth.

## 2 Materials and methods

### 2.1 Ligand docking to NKA

All stable EO conformations were obtained earlier using the RHF/6-31G* method (Plakhova et al., 2020), while the possible conformations of the ouabain–Na^+^ chelate complex (OUA-Na) are obtained herein in accordance with the same protocol. The ligand structures where the A ring adopted not the chair but the twist conformation were taken out of further consideration because they were previously shown not to dock with NKA effectively (Rogachevskii et al., 2023). The Ca^2+^ cation is chelated either by five (O^1^, O^3^, O^5^, O^19^, O^5^′) or by three (O^1^, O^11^, O^19^) oxygen atoms (Figures 1C,D). These chelation modes are designated further as EO5 and EO3, respectively. In addition to that, there are two ways of orientation of the lactone ring E with respect to the steroid core, which brings the total count of stable EO conformations to four. Only one mode of Na^+^ chelation and two corresponding conformations differing by the E ring orientation have been identified, the cation being chelated by three (O^1^, O^5^, O^19^) oxygen atoms (Figure 1E).

The pig ouabain-sensitive α1-NKA isoform (α1S-NKA) was obtained from PDB (PDB code 4HYT) (Laursen et al., 2013). Using the PyMOL (Schrödinger and DeLano, 2020) standard tools, the rat ouabain-resistant NKA α1-subunit (α1R-NKA) was constructed from α1S-NKA substituting Gln111 and Asn122 in the initial structure for Arg111 and Asp122. This substitution is the factor that mainly accounts for the ouabain resistance of rodent α1-NKA (Lingrel, 2010). After hydrogens were added to heavy atoms using AutoDockTools (Sanner, 1999), the entire NKA structures were fully optimized using the gradient descent method in the UFF forcefield (Rappé et al., 1992) in the framework of OpenBabel 2.4 program package. Local docking was performed within the cubic 50 Å box centered at the bound ouabain geometry center available from the original structure, 5 times for each ligand conformation, using AutoDock Vina (Trott and Olson, 2010) in accordance with the earlier protocol (Rogachevskii et al., 2023). The ligand–NKA complexes were once again minimized after docking. The obtained data were analyzed with PyMOL (Schrödinger and DeLano, 2020), which has also been used to produce the structure images of the ligands and ligand-NKA complexes. The hydrogen bonds and salt bridges were detected manually, the distance between heavy atoms in a bond did not exceed 4 Å.

### 2.2 Animal subject ethical approval

Institutional Review Board Statement: Experiments were designed in accordance with the European Communities Council Directive of 24 November 1986 (86/609/EEC). The Local Committee for Animal Care and Use at Pavlov Institute of Physiology of the Russian Academy of Sciences approved all experimental procedures with the animals, permit number 12/09/2021 (12 December 2021). Animals were treated following the Guide for the Care and Use of Laboratory Animals (8th edition, National Academies Press). All animals were obtained from the Biocollection of Pavlov Institute of Physiology of the Russian Academy of Sciences.

### 2.3 Formalin test

The classical formalin test is widely used to study the different mechanisms of analgesia induced by various new medicinal substances and the acute to chronic pain transition (Price et al., 2018; Zhang et al., 2018). Its advantage over other models of inflammatory pain is that it allows to simulate both acute and tonic pain using only one substance, the chemical irritant formalin, over a relatively limited time (60 min) (Dubuisson and Dennis, 1977; Tjølsen et al., 1992). The organization of these types of pain in two phases with the interphase between them is a unique feature of the test. In the formalin test, the subcutaneous injection of formalin into the pad of a hind limb of the rat evokes the specific types of pain-related behavior, flexing, shaking and licking, alternating with each other. Flexing and shaking are organized at the spinal level, licking, at the supraspinal level. The first short acute phase (Ph1, 1–5 min) is the response of peripheral nociceptive system to the formalin injection, and reflects the acute pain. Then the interphase (6–9 min), a period of quiescence, follows. The second prolonged phase (Ph2, 12–40 min) is caused by the developing inflammatory process and sensitization induced by the first phase, and reflects the tonic pain.

The study was carried out on adult *Wistar* male rats. Four experienced assistants, who have been conducting these experiments for many years, performed the formalin test. The animals (300–360 g) were divided into three groups. The first group was subjected to the administration of EO (*n* = 7); the second group, OUA-Na, the ouabain–Na^+^ chelate complex containing Na^+^ instead of Ca^2+^ (*n* = 7); the control group, physiological saline (*n* = 6). The EO and OUA-Na solutions were obtained preincubating ouabain for 24 h in distilled water with CaCl_2_ or NaCl, correspondingly, at the concentrations three orders of magnitude higher than that of ouabain. Ten minutes prior to the formalin injection (2.5%, 50 μL, subcutaneously into the pad of the left hind limb), the EO and OUA-Na solutions (0.3 mg/kg, 1 mL, intraperitoneally) were administered to the corresponding groups of experimental rats, the same volume of physiological saline was administered to the control rats. The animals were then placed in a chamber to register the flexing+shaking behavior (the spinal level) and the licking duration of the formalin-injected limb (the supraspinal level) within 60 min using the hardware-software setup.

A preliminary analysis of the data was carried out using the Wilcoxon Signed Ranks test to compare the first and second phase of the experiment, as well as the Mann-Whitney test to compare experimental and control data separately in each phase. After that, the data were analyzed using mixed dispersion analysis with factors: phase (first and second) and exposure (control, OUA-Na, and EO) with subsequent testing of simple effects. The analysis was conducted separately for licking and flexing/shaking. Differences were considered statistically significant at *p* < 0.05. The calculations were performed using the IBM SPSS Statistics 26 software complex. The data are presented as the mean value ± SEM. The methodological quality of the study in the formalin test was assessed according the ARRIVE-style details (randomization, blinding, exclusion criteria). Rats were randomly assigned to the experimental groups, given that random assignment ensures an unbiased result. Blinding conditions were always observed. There were no exclusions in these experiments.

### 2.4 Organotypic nerve tissue culture method

DRG explants were obtained from 10 to 12 days old *White Leghorn* chick embryos as described before (Penniyaynen et al., 2019; Plakhova et al., 2020; Rogachevskii et al., 2022) and cultured in the medium consisting of Hank’s solution (45%), Eagle’s minimal essential medium (40%), 10% fetal bovine serum (FBS), insulin (0.5 U/mL), glucose (0.6%), L-glutamine (2 μM) and gentamicin (100 U/mL) in a humidified incubator (Sanyo, Osaka, Japan) with 5% CO_2_ at 37 °C. Ouabain, pifithrin-α, and JSH-23 were added to the culture medium at 0.1 nM, 0.1 μM, and 1 μM, respectively. All reagents used were from Sigma (Sigma-Aldrich, St. Louis, MA, United States). The control explants were cultured without any of the three studied substances. All explants were visualized using the inverted optical Axio Observer Z1 microscope (Carl Zeiss, Oberkochen, Germany) after 3 days of culturing. The obtained images were analyzed with ImageJ (National Institutes of Health, Bethesda, MD, United States) and ZEN_2012 (Carl Zeiss) software. The area index (AI) was calculated as the ratio of the peripheral growth zone area to the central zone area, and its average value in the control explants was taken as 100%. Experiments were conducted using the equipment of the Confocal Microscopy Collective Use Center at the Pavlov Institute of Physiology of the Russian Academy of Sciences.

### 2.5 Immunofluorescence staining

Immunofluorescent staining was performed as described previously (Plakhova et al., 2020; Rogachevskii et al., 2022). DRG explants were fixed in freshly made 4% paraformaldehyde at room temperature for 3 min and then permeabilized with 0.3% Triton X-100. After blocking with 10% FBS at room temperature for 30 min, the explants were incubated with the anti-growth associated protein 43 (GAP-43) antibody overnight at 4 °C. Next, the explants were washed with phosphate-buffered saline three times and incubated with TRITC-conjugated secondary antibody for 2 h at room temperature. The images were captured with the confocal laser scanning microscope LSM-710 (Carl Zeiss) integrated with the Axio Observer Z1 microscope (1,024 ×1,024 resolution; A-Plan 10×/0.25 lens).

### 2.6 Confocal laser scanning microscopy and immunofluorescence image analysis

The immunofluorescence intensity of the antibodies to GAP-43 (IfiGAP43) was evaluated with the LSM-710 microscope using ZEN_2012 software. This software allows for the image acquisition and quantitative analysis of immunofluorescence intensity (Ando et al., 2016; Fukuchi et al., 2015; Plakhova et al., 2020; Pyeon and Lee, 2012; Ren et al., 2016; Son et al., 2019). Optical density measurements were carried out in the immunofluorescence-stained section. ZEN_2012 software made it possible to locate the areas where IfiGAP43 obeyed the law of Gaussian distribution. Non-specific binding of antibodies obeyed the exponential distribution law. When the latter process strongly affected the Gaussian distribution, the data were neglected to ensure the accuracy and reliability of IfiGAP43 measurements. The mean IfiGAP43 value in the control explants was taken as 100%.

### 2.7 Statistical analysis

The statistical analysis of the data obtained by all experimental methods other than the formalin test was performed using the STATISTICA 10.0 software (StatSoft, Inc., Tulsa, OK, United States). The samples were tested for normal distribution using the Shapiro-Wilk test. The data met the criteria, enabling us to assess the significance of differences between the control and experimental data using Student’s *t*-test. For comparing the control and experimental groups, Student’s *t*-test for two independent samples was applied. The results are presented as the mean value ± SEM. Differences were considered statistically significant at *p* < 0.05.

## 3 Results

### 3.1 Docking with NKA

The structural features of the pig ouabain-sensitive α1-NKA (α1S-NKA) and the rat ouabain-resistant α1-NKA (α1R-NKA) were discussed in detail earlier (Rogachevskii et al., 2023). The spatial positions of the amino acid residues directly contacting the studied ligands mainly overlap in two models. The only noticeable change is that the Gln111–Asn122 hydrogen bond detected in α1S-NKA is substituted with the Arg111–Glu116 ionic bridge in α1R-NKA. The binding site is comprised of 21 residues (Gln/Arg111, Glu116, Glu117, Pro118, Asp121, Asn/Asp122, Leu125, Glu312, Ile315, Phe316, Gly319, Val322, Ala323, Glu327, Phe783, Phe786, Leu793, Thr797, Ile800, Arg880, Asp884), 10 of which are polar. Except for Glu116, all polar residues are involved in intermolecular hydrogen bonds with the free ouabain molecule (OUA) in the OUA–NKA complex (Rogachevskii et al., 2023).

**FIGURE 2:**
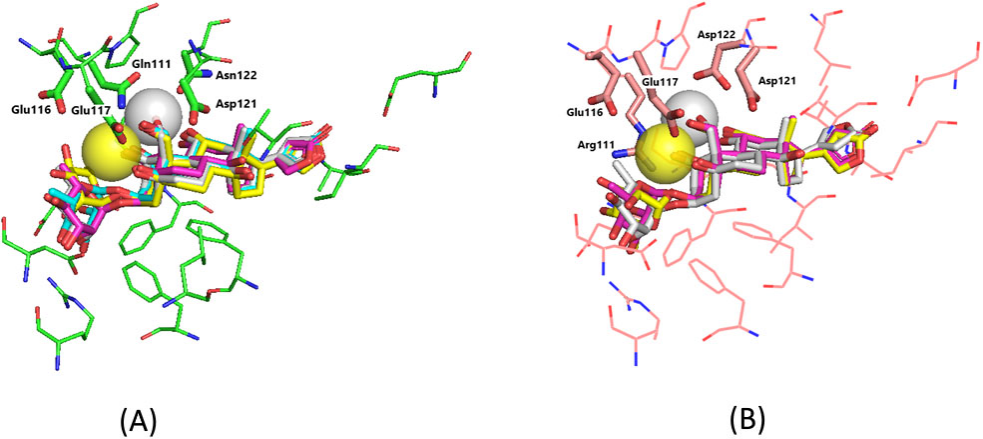
Superimposition of the docked ligands in their NKA binding site. **(A)** α1S-NKA. OUA, gray sticks (carbon, gray; oxygen, red); EO5, cyan sticks (carbon, cyan; oxygen, red; Ca^2+^, yellow transparent sphere); EO3, yellow sticks (carbon, yellow; oxygen, red; Ca^2+^, gray transparent sphere); OUA-Na, magenta sticks (carbon, magenta; oxygen, red; Na^+^ position almost coincides with that of Ca^2+^ in EO5 and is not shown in the figure). α1S-NKA is presented with green lines (carbon, green; oxygen, red; nitrogen, blue). The Gln111, Glu116, Glu117, Asp121, and Asn122 side chains are highlighted with green sticks. RMSD = 0.243 Å. **(B)** α1R-NKA. OUA, gray sticks (carbon, gray; oxygen, red); EO5, cyan sticks (carbon, cyan; oxygen, red; Ca^2+^, yellow transparent sphere); EO3, yellow sticks (carbon, yellow; oxygen, red; Ca^2+^, gray transparent sphere). RMSD = 0.314 Å. α1R-NKA is presented with pink lines (carbon, pink; oxygen, red; nitrogen, blue). The Arg111, Glu116, Glu117, Asp121, and Asp122 side chains are highlighted with pink sticks. OUA positions were taken from our prior publication (Rogachevskii et al., 2023). Hydrogen atoms are not shown. The steroid cores of all ligands dock essentially at the same position, while the rhamnosyl rings display some conformational lability.

Docking of EO5, EO3, and OUA-Na with both NKA models has shown that the ring E orientation has no substantial effect on the ligand position. Quite unexpectedly, OUA-Na has not been found to effectively dock with α1R-NKA in the correct orientation. Superimposition of the docked ligands is displayed in Figure 2. The steroid cores (rings A-D, Figure 1A) of all studied ligands dock essentially at the same depth within their NKA binding site. Structural parameters of the obtained ligand–NKA complexes are presented in Table 1.

**TABLE 1:** Characteristics of ligand–NKA complexes obtained by docking.

NKA isoform	α 1S-NKA				α 1R-NKA		
Ligand	OUA	EO5	EO3	OUA-Na	OUA	EO5	EO3
Hydrogen bonds formed between the steroid core and NKA	7	7	7	7	5	5	5
Hydrogen bonds formed between the rhamnosyl ring and NKA	5	7	4	5	5	7	3
Predicted binding energy to NKA, kcal/mol, E_b_	−11.0	−12.4	−10.5	−11.6	−9.5	−10.6	−9.9
Ligand-NKA complex association constant, mol^–1^, K_a_	1.1 × 10^8^	1.2 × 10^9^	5.0 × 10^7^	3.2 × 10^8^	9.1 × 10^6^	5.9 × 10^7^	1.8 × 10^7^

The steroid core oxygens form seven hydrogen bonds with α1S-NKA and only five hydrogen bonds with α1R-NKA, exactly as what has been observed earlier for OUA (Rogachevskii et al., 2023). To sterically accommodate the chelated cation in the binding site, the relatively unconstrained O^19^ oxygen atom significantly shifts its position as compared with that upon OUA binding. As a consequence, the Asn/Asp122–O^19^ hydrogen bond present in the OUA–NKA complexes is substituted with the Glu117–O^19^ hydrogen bond. Other intermolecular hydrogen bonds formed by the steroid core of OUA remain intact, and their total number is not affected by the chelated cations upon docking of EO5, EO3, and OUA-Na.

However, the cation chelation noticeably affects the patterns of hydrogen bonds formed by the rhamnosyl rings that dock closer than the steroid cores to the NKA extracellular surface. Because the binding pocket is relatively wide there, several modes of the rhamnosyl ring docking have been detected in the ligand–NKA complexes (Figure 2). Accommodation of the Ca^2+^ cation in the EO3–NKA complexes slightly distorts the A ring conformation, which rotates the rhamnosyl ring so that it forms less hydrogen bonds with NKA than in the OUA–NKA complex (Table 1). In the EO5–NKA complexes, the O^3^ oxygen atom that links the rhamnosyl ring to the steroid core and the rhamnosyl O^5^′ oxygen atom directly participate in the Ca^2+^ chelation. Hence, the rhamnosyl ring becomes orientated in such a way that it can form two additional hydrogen bonds with NKA. The O^3^ and O^5^′ oxygen atoms are not involved in the Na^+^ chelation, and the number of hydrogen bonds formed by the rhamnosyl rings of OUA-Na and OUA upon binding to NKA is the same.

Upon EO5 docking with both NKA isoforms, the Ca^2+^ double positive charge is completely compensated by intermolecular ionic bonds with the Glu116 and Glu117 carboxylate anions located within 4 Å from the bound cation. These two anions should also be responsible for the compensation of the Na^+^ single positive charge after OUA-Na binding. However, one of the anions requires a positively charged counterion to keep the local charge equal to zero. Finally, there are no carboxylate NKA anions detected close to the chelated Ca^2+^ in the EO3–α1S-NKA complex, the least distant being the Asp121 carboxylate anion at 5 Å from the bound cation, so the cation double positive charge is not electrostatically compensated. In α1R-NKA, the Asn/Asp122 substitution allows for a partial compensation of the Ca^2+^ charge by the Asp122 carboxylate anion.

The prior observations correlate well with the predicted NKA binding energies (E_*b*_) of the studied ligands (Table 1). The complete compensation of the Ca^2+^ double positive charge in the EO5–NKA complexes makes the EO5 binding with α1S-NKA and α1R-NKA 1.4 and 1.1 kcal/mol more energetically favorable than that of OUA, respectively. The OUA-Na binding to α1S-NKA is 0.6 kcal/mol more energetically favorable than that of OUA. In the EO3–α1S-NKA complex, the Ca^2+^ double positive charge is not electrostatically compensated at all, which makes the EO3 binding with α1S-NKA 0.5 kcal/mol less favorable than that of OUA. A partial compensation of the Ca^2+^ charge by the Asp122 carboxylate anion in the EO3–α1R-NKA complex has the energetic effect of 0.4 kcal/mol. The association constants K_*a*_ of the ligand–NKA complexes were calculated from the following expression: E_*b*_ = -2.3RTlgK_*a*_, where R is the molar gas constant, and T is the absolute temperature. At 25°C, E_*b*_ = -1.31lgK_*a*_ (kcal/mol). According to the conventional classification, the ligand–receptor complexes with the K_*a*_ values in the range of 10^6^–10^9^ mol^–1^ are considered moderately stable to stable. This is the case for all ligand–NKA complexes studied herein due to the high value of the steroid core binding energy.

**FIGURE 3:**
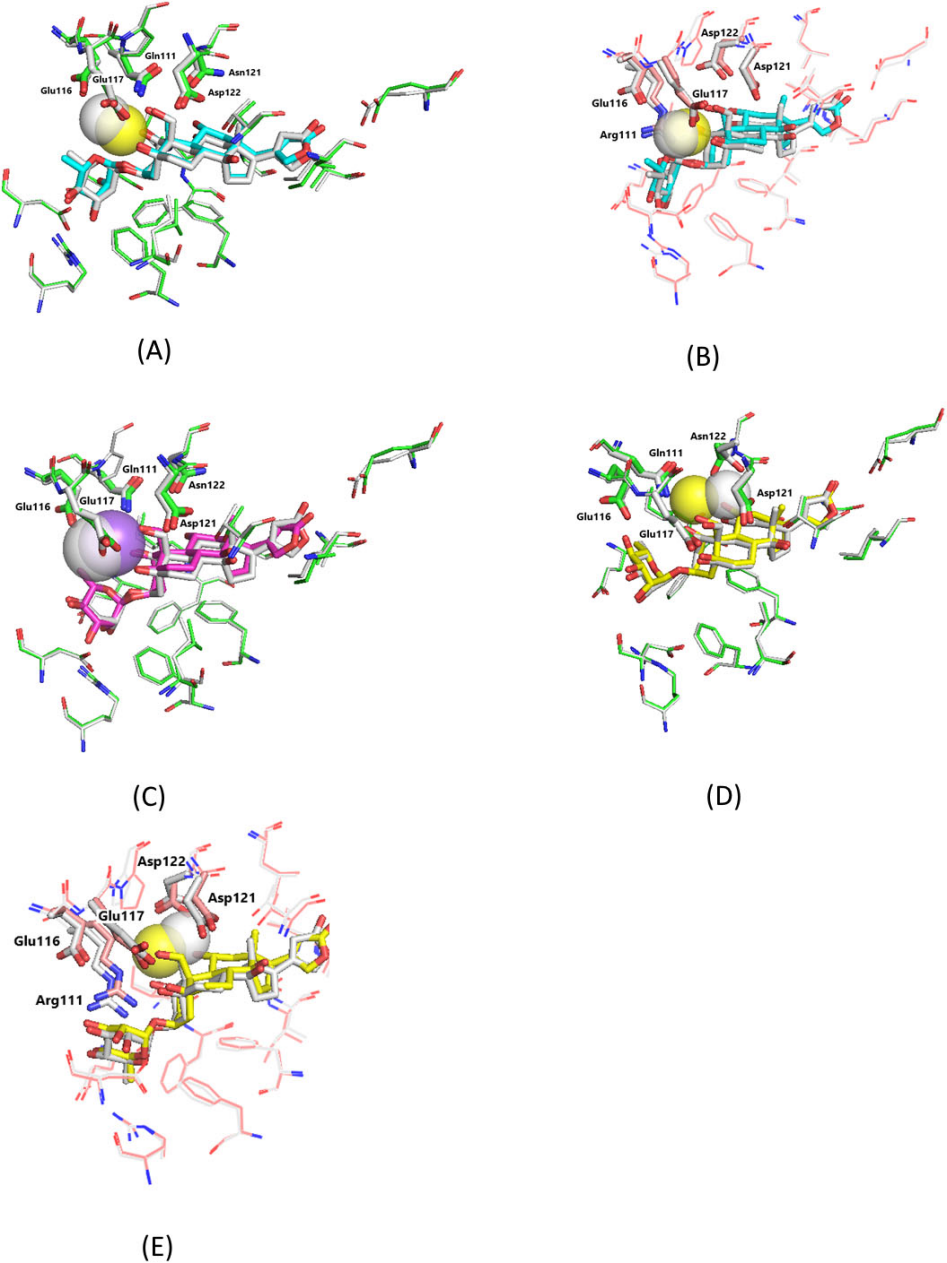
Superimposition of the ligand binding sites in the ligand–NKA complexes before and after geometry optimization. **(A)** EO5–α1S-NKA. Before optimization, EO5, cyan sticks (carbon, cyan; oxygen, red); Ca^2+^, yellow transparent sphere; α1S-NKA, green lines (carbon, green; oxygen, red; nitrogen, blue). After optimization, EO5, gray sticks (carbon, gray; oxygen, red); Ca^2+^, gray transparent sphere; α1S-NKA, gray lines (carbon, gray; oxygen, red; nitrogen, blue). **(B)** EO5–α1R-NKA. Before optimization, EO5, cyan sticks (carbon, cyan; oxygen, red); Ca^2+^, yellow transparent sphere; α1R-NKA, pink lines (carbon, pink; oxygen, red; nitrogen, blue). After optimization, EO5, gray sticks (carbon, gray; oxygen, red); Ca^2+^, gray transparent sphere; α1R-NKA, gray lines (carbon, gray; oxygen, red; nitrogen, blue). **(C)** OUA-Na–α1S-NKA. Before optimization, OUA-Na, magenta sticks (carbon, magenta; oxygen, red); Na^+^, violet transparent sphere; α1S-NKA, green lines (carbon, green; oxygen, red; nitrogen, blue). After optimization, OUA-Na, gray sticks (carbon, gray; oxygen, red); Na^+^, gray transparent sphere; α1S-NKA, gray lines (carbon, gray; oxygen, red; nitrogen, blue). **(D)** EO3–α1S-NKA. Before optimization, EO3, yellow sticks (carbon, yellow; oxygen, red); Ca^2+^, yellow transparent sphere; α1S-NKA, green lines (carbon, green; oxygen, red; nitrogen, blue). After optimization, EO3, gray sticks (carbon, gray; oxygen, red); Ca^2+^, gray transparent sphere; α1S-NKA, gray lines (carbon, gray; oxygen, red; nitrogen, blue). **(E)** EO3–α1R-NKA. Before optimization, EO3, yellow sticks (carbon, yellow; oxygen, red); Ca^2+^, yellow transparent sphere; α1R-NKA, pink lines (carbon, pink; oxygen, red; nitrogen, blue). After optimization, EO3, gray sticks (carbon, gray; oxygen, red); Ca^2+^, gray transparent sphere; α1R-NKA, gray lines (carbon, gray; oxygen, red; nitrogen, blue). The Gln/Arg111, Glu116, Glu117, Asp121, and Asn/Asp122 side chains are highlighted with the sticks of the corresponding colors. Hydrogen atoms are not shown.

It should be noted that the entire NKA structures were minimized before docking, but they were considered rigid in the process of ligand docking. To take the conformational effects into consideration, the obtained ligand–NKA complexes were once again optimized after docking. Superimposition of the ligand binding sites in the ligand–NKA complexes before and after optimization is displayed in Figure 3. The total number of intermolecular hydrogen bonds with NKA remains the same after optimization for all studied ligands, as well as the orientation of the NKA amino acid residues side chains. In the EO5–NKA complexes, the Ca^2+^ cation shifts ∼1 Å toward the Glu116 carboxylate anion (Figures 3A,B) without breaking any of the five intramolecular Ca–O chelating bonds. A similar shift of the Na^+^ cation is observed in the OUA-Na–α1S-NKA complex (Figure 3C), the three Na–O chelating bonds are retained but they weaken considerably. In the EO3–NKA complexes, the integrity of the NKA-bound ligand is lost after subsequent optimization; the Ca^2+^ cation shifts ∼2 Å toward the Asp121 carboxylate anion, which is accompanied by the cleavage of the Ca–O^1^ chelating bond in both models and results in a significant rotation of the Asn122 side chain in α1S-NKA required to accommodate the cation in the new position (Figures 3D,E).

The geometry changes after the optimization of the ligand–NKA complexes obtained by docking correlate well with the required coordination number of the chelated Ca^2+^ cations. The usual coordination number for the Ca^2+^ cation in aqueous solution is 6, though in some cases, especially in the crystal lattice, the coordination numbers of up to 12 were also detected. Given the 1:1 steroid–cation stoichiometry, the cation coordination numbers in the initial ligand molecules, EO5 and EO3, are 5 and 3, correspondingly, which is less than 6. In the optimized EO5–NKA complexes (Figures 3A,B), the chelated Ca^2+^ is bound by 7 oxygens: 5 chelating bonds within the ligand and two intermolecular ionic bonds with the Glu116 and Glu117 carboxylate anions, which provide at least one oxygen each. However, the side chain Glu116 and Glu117 conformations, where all four oxygens bind the cation are sterically allowed, which could potentially increase the Ca^2+^ coordination number to 9. In the optimized EO3–NKA complexes (Figures 3D,E), the cation is unambiguously coordinated by 6 atoms: two ouabain oxygens (O^11^ and O^19^), and four non-carbon side chain atoms of the Asn/Asp121 and Asp122 NKA amino acid residues.

As long as the results presented herein describe the ligand docking into the previously known NKA binding pocket, we find it excessive to present the data on the entire NKA structures. This information would be relevant if we aimed to discuss the further conformational changes triggered by the EO binding, which is beyond the scope of the current study. Because we have not visually detected any noticeable structural changes between the superimposed NKA structures before and after energy minimization, the global root mean square deviation (RMSD) values are also excessive for the understanding of our mechanistic model. The results obtained indicate that EO5 is the physiologically relevant form of EO.

### 3.2 Formalin test

**FIGURE 4:**
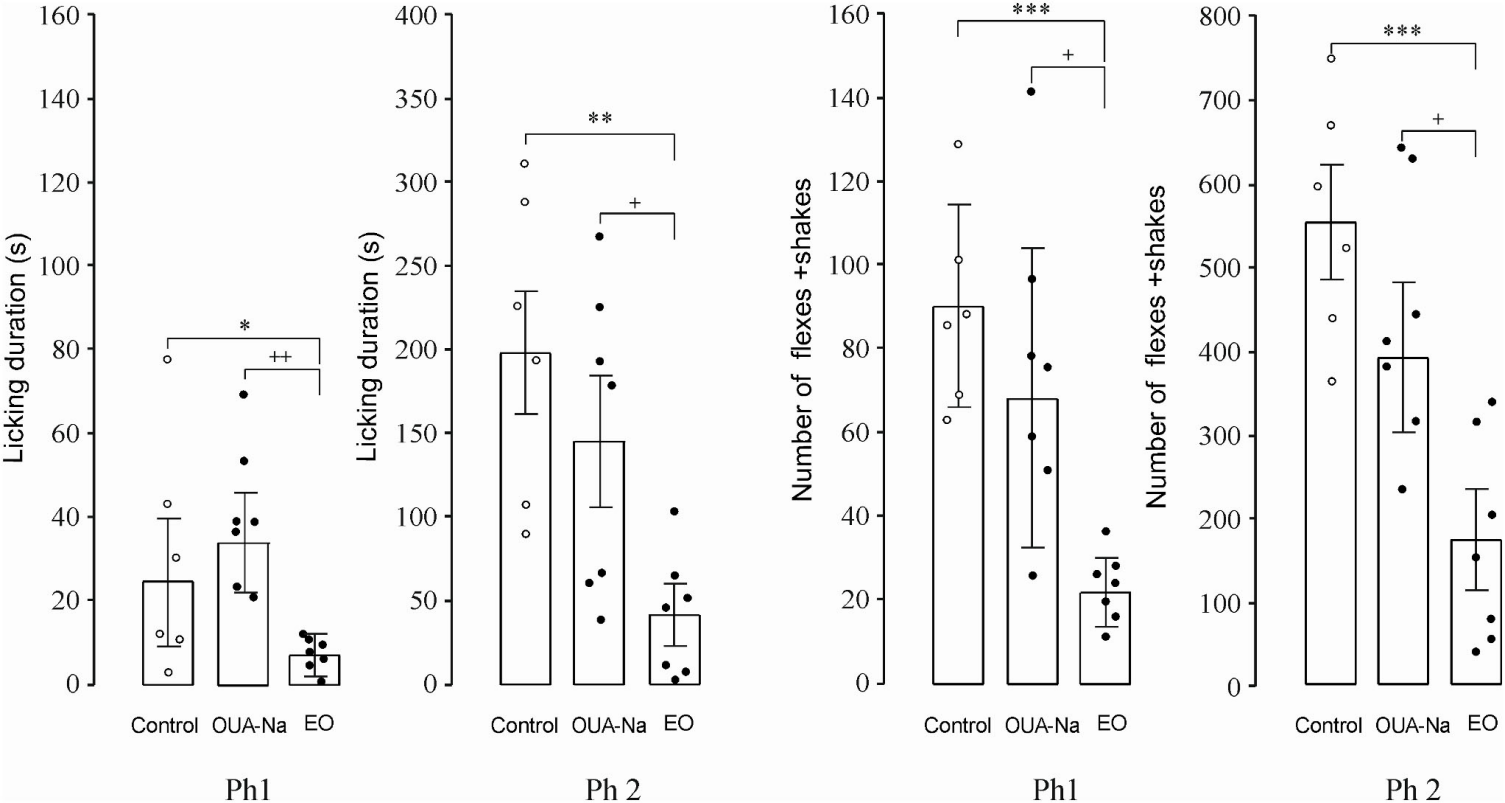
Effects of EO (*n* = 7) as compared to control (*n* = 6) and OUA-Na (*n* = 7) on the licking duration and the number of flexes+shakes in the first acute (Ph1) and second tonic (Ph2) phases of the formalin test. *p < 0.05, **p < 0.01, ***p < 0.001 EO vs. controls. +*p* < 0.05, ++*p* < 0.01 EO vs. OUA-Na. EO and OUA-Na were injected intraperitoneally (0.3 mg/kg, 1 mL).

The data obtained after the EO injection show that the licking duration (the supraspinal level) during the first acute phase (Ph1) is significantly lower as compared with the control (8.6 ± 1.6 vs. 30.0 ± 11.4 s, *p* = 0.049) and OUA-Na (8.6 ± 1.6 vs. 35.1 ± 8.1 s, *p* = 0.003). The licking duration during the second tonic phase (Ph2) is also significantly lower as compared with the control (44.8 ± 17.3 vs. 196.8 ± 37.8 s, *p* = 0.049) and OUA-Na (44.8 ± 17.3 vs. 147.0 ± 34.2 s, *p* = 0.020). The EO injection reduces the number of flexes+shakes (the spinal level) during Ph1 as compared with the control (24.3 ± 3.1 vs. 95.7 ± 11.7 s, *p* = 0.049) and OUA-Na (24.3 ± 3.1 vs. 79.6 ± 21.5 s, *p* = 0.025); during Ph2, the EO administration results in a similar response as compared with the control (175.0 ± 49.3 vs. 561.5 ± 58.4 s, *p* < 0.0001) and OUA-Na (175.0 ± 49.3 vs. 387.4 ± 81.5 s, *p* = 0.045). Thus, the data presented in Figure 4 demonstrate that the administration of EO at 0.3 mg/kg evokes an antinociceptive effect, as opposed to the administration of OUA-Na at the same very low dosage.

### 3.3 Organotypic tissue culture

**FIGURE 5:**
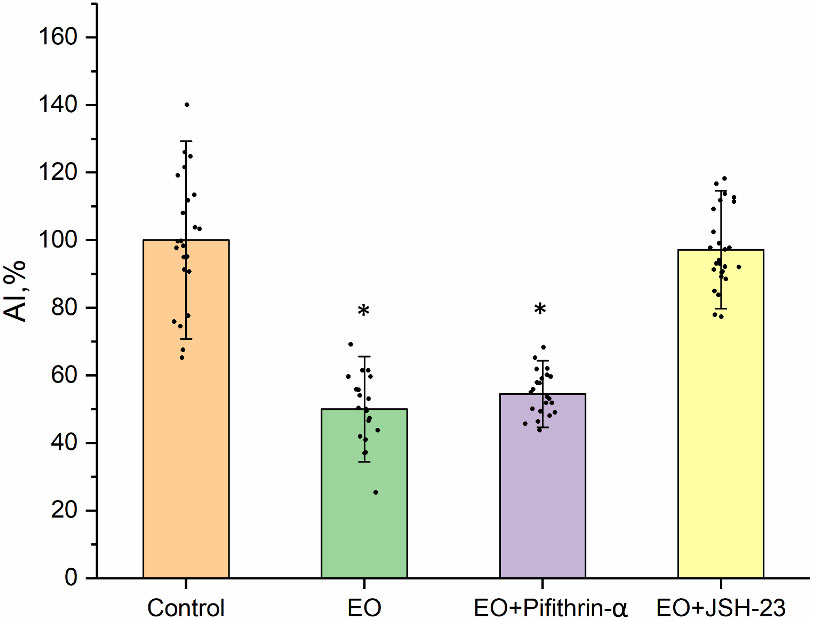
The effect of EO on neurite growth in DRG explants in the presence of inhibitors of transcription factors p53 (pifithrin-α, 0.1 μM) and NF-kB (JSH-23, 1 μM). The ordinate axis—area index (AI, %). Data are presented as mean ± SEM. *p < 0.05.

To test the involvement of the p53 transcription factor in the EO-triggered NKA/Src/PKA/p38 MAPK signaling cascade that controls the EO neurite-inhibiting effect, its specific inhibitor pifithrin-α (0.1 μM) has been added to the culturing medium 20 min before EO (0.1 nM). The EO neurite-inhibiting effect has not been blocked by the p53 inhibitor (Figure 5). The AI value upon combined application of EO and pifithrin-α is 50 ± 5% (*n* = 25, *p* < 0.05) less than the control (*n* = 23), same as observed earlier for EO alone (Plakhova et al., 2020). Hence, the p53 transcription factor is not involved in the EO-triggered downstream signaling in the nociceptive neuron.

When JSH-23 (1 μM), the selective inhibitor of the NF-κB transcription factor, was added to the culturing medium 20 min prior to EO (0.1 nM), the EO neurite-inhibiting effect disappeared completely. The AI of the explants treated with EO and JSH-23 together (*n* = 28) was the same as in the control (*n* = 23), indicating that NF-κB is involved in the EO-triggered NKA/Src/PKA/p38 MAPK signaling cascade (Figure 5).

**FIGURE 6:**
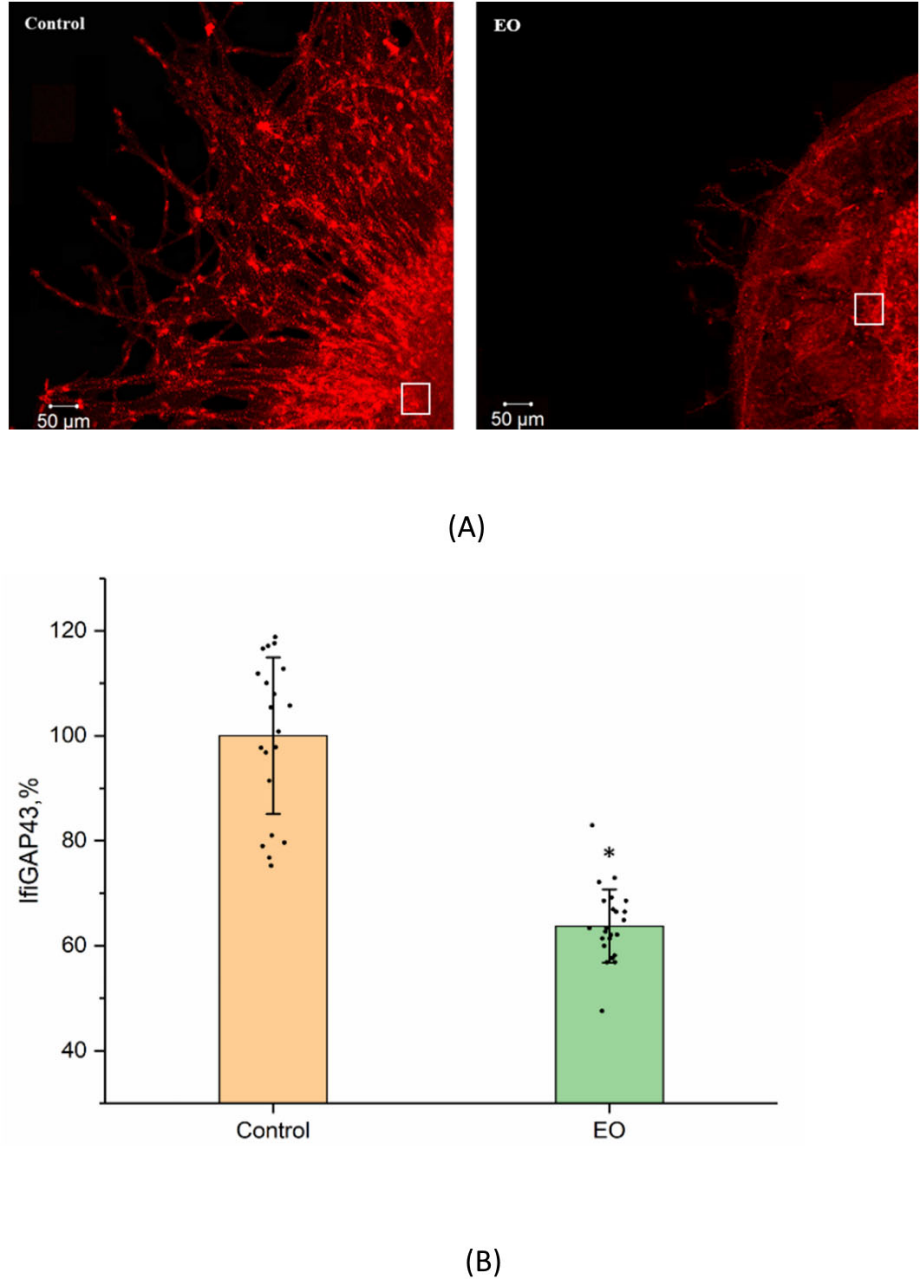
Effects of EO on neurite growth and GAP-43 expression in the nociceptive neuron. **(A)** Fragments of DRG explant growth zones in the control conditions (left) and after EO application at 0.1 nM (right). Both control and EO-treated neurons were immunostained with the antibodies to the GAP-43 protein (red), ×10. The white box marks the selected area (50 × 50 μm) involving neurites and cell bodies. Ten such areas of the same size were chosen in each of the control (*n* = 20) and EO-treated (*n* = 22) explants to measure the immunofluorescence intensity of the antibodies to GAP-43, IfiGAP43. **(B)** Decrease in IfiGAP43 after EO application. The ordinate axis—immunofluorescence intensity (IfiGAP43, %). Data are presented as mean ± SEM. *p < 0.05.

To determine whether the GAP43 gene may be the effector unit that regulates the EO-triggered inhibition of neurite growth, the antibodies to the GAP-43 protein were applied. The captured images presented in Figure 6A demonstrate that EO decreases the GAP-43 protein production and inhibits the neurite growth. The significant decrease (45 ± 7%, *n* = 22, *p* < 0.05) in the immunofluorescence intensity of the GAP-43 antibodies (IfiGAP43) has been observed in the nociceptive neuron after the EO (0.1 nM) application as compared with the control (*n* = 20) (Figure 6B).

## 4 Discussion

The current study is aimed to further elucidate the physiological role of EO–NKA binding and EO-triggered NKA signaling in the nociceptive neuron identified as such due to the presence of the Na_*V*_1.8 channels in its membrane (Bennett et al., 2019). The distribution of the Nav1.8 channels across all neurites of sensory neurons in chick embryos has been revealed earlier using immunocytochemical methods (Plakhova et al., 2020), which justifies that the neurons used in the present experiments are considered nociceptive.

**FIGURE 7:**
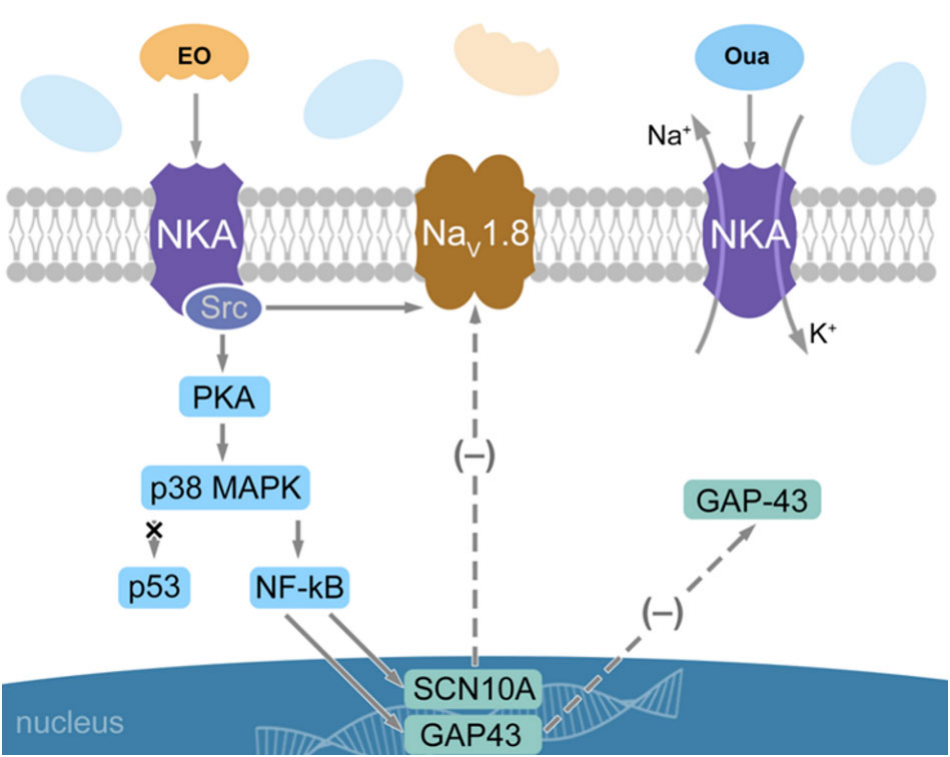
Scheme illustrating the EO-triggered Na,K-ATPase (NKA) signaling in the nociceptive neuron. EO is the Ca^2+^ chelate complex of the ouabain molecule. When the EO binding triggers the NKA signaling function, three different pathways are activated. The tangential signaling along the neuron membrane from NKA to the Na_V_1.8 channel evokes a rapid decrease in the effective charge of the Na_V_1.8 channel activation gating system. The downstream NKA/Src/PKA/p38 MAPK/NF-κB signaling modulates the expression of two different genes, SCN10A and GAP43 (solid lines). This delayed process results in a decrease in the density of the Na_V_1.8 channels in the neuron membrane (via SCN10A) and in an inhibition of DRG neurite growth (via GAP43) which is also manifested in a decrease in the GAP-43 protein production (dashed lines, marked with a minus sign in brackets). Concentrations of EO triggering the NKA signaling are in the nanomolar range, which is several orders of magnitude less than required to control the NKA pumping function. The NKA molecule on the right implements its pumping function, which is not under EO control. GAP-43—Growth-associated protein-43; PKA—protein kinase A; p38 MAPK—p38 mitogen-activated protein kinase; NF-κB—the nuclear factor κB.

Mathematical modeling of the Na_*V*_1.8 channel functional activity in the case when the effective charge transferred by the Na_*V*_1.8 channel activation gating system is the only changing parameter of the model has demonstrated the following (Skrebenkov et al., 2023). If the effective charge is decreased to a certain value as compared with the control, the Nav1.8 channel voltage sensitivity is reduced to such an extent that the impulse firing of the nociceptive neuron is completely restored back to the normal stimulus-response function. Only the high-frequency component has been specifically eliminated from its membrane response, which provides the basis for the antinociceptive effect of a substance that might sufficiently decrease the effective charge (Krylov et al., 2017). The same effect has been also achieved by reducing the Na_*V*_1.8 channel density in the nociceptive neuron membrane. Our prior electrophysiological patch-clamp research has demonstrated that the EO application at 10 nM significantly decreases the Na_*V*_1.8 channel activation gating system effective charge (Plakhova et al., 2020), thus making EO a modulator of the Na_*V*_1.8 channel functional activity, as opposed to the Na_*V*_1.8 channel blockers that reduce the density of the active channels in the membrane. However, the effect of potential analgesics will apparently be less specific in the latter case because of their possible interactions with other members of the voltage-gated sodium channel superfamily. In addition to that, EO also modulates the expression of the SCN10A gene and decreases the production of Na_*V*_1.8 channels which could otherwise counterbalance their blocking (Figure 7). Due to a unique combination of its chemical structure and the ability to chelate Ca^2+^, EO is rather likely the only naturally occurring analgesic cardiotonic steroid.

The data presented herein are consistent with the NF-κB involvement in the EO signaling, which is likely the factor responsible for the bifurcation of the EO-triggered NKA downstream signaling pathways. This transcription factor regulates the expression of multiple genes that control a number of cellular processes such as proliferation, inflammation, differentiation, survival, and migration (Dolcet et al., 2005; Moynagh, 2005). It also plays an important role in the development and pathogenesis of the nerve tissue. Activation of the NF-κB-dependent signaling mediated by neurotrophic factors facilitates the survival of neurons, controls the neurite growth, myelin production, and axon regeneration (Blank and Prinz, 2014; Gutierrez et al., 2005; Mincheva-Tasheva and Soler, 2013). It has been also shown that NF-κB participates in nociception and regulates the pathogenesis of neuropathic pain by the expression of inflammatory mediators (Sakaue et al., 2001). Suggestively, it is at the NF-κB level that the EO-triggered NKA downstream signaling cascade bifurcates; one of the pathways modulates the SCN10A gene, while the other pathway decreases the GAP43 gene expression (Figure 7). An essential supporting argument is the decrease in the GAP-43 protein production associated with the GAP43 gene expression. This protein controls the formation of nervous connections, their regeneration and plasticity (Frey et al., 2000; Holahan, 2015). A decrease in the GAP-43 production mediated by the NKA/Src/PKA/p38 MAPK/NF-κB signaling pathway results in the DRG neurite growth inhibition at the tissue level (Figure 6). Our data also indicate that another transcription factor, p53, is not involved in the EO-triggered NKA downstream signaling.

The obtained results (Figures 5, 6) suggest a new endogenous mechanism for modulating the NF-κB functional activity, which is of major importance for the creation of new drugs. It is known that the transcription factor NF-κB is used as a target molecule for the development of anti-inflammatory, antitumor and proapoptotic drugs. The conventional approach is based on the use of exogenous substances that directly bind to this protein and thus modulate its functioning, and the criterion for their selection is the theoretically calculated binding energy, since the three-dimensional structure of the protein is well known (Piccagli et al., 2008). A remarkable advantage of our approach is that NF-κB is modulated indirectly via the NKA downstream signaling. Endogenous attacking molecule applied in the nanomolar range of concentrations, EO, triggers the NKA signaling function in the nociceptive neuron. Activation of the NKA/Src/PKA/p38 MAPK/NF-κB downstream cascade modulates the NF-κB functioning by this physiological mechanism, which solves the problem of delivering other exogenous molecules to this target.

The docking studies made it possible to elucidate the critical role of the chelated Ca^2+^ cation in the process of EO-NKA binding. The chelated cation structurally differentiates EO from OUA, therefore, it should determine the ability of EO to trigger the NKA signaling as opposed to OUA. The results obtained favor the idea that EO5, the ouabain–Ca^2+^ chelate complex where the cation is coordinated by five oxygen atoms (Figure 1C), is the physiologically relevant EO conformation. It is demonstrated that the steric accommodation of the cations in all ligand-NKA complexes studied herein does not substantially affect the hydrogen bond network formed between the steroid core of the ligands and NKA, which provides the main contribution to the predicted ligand-NKA binding energies. However, the Ca^2+^ chelation affects the binding of the rhamnosyl ring that has been found necessary for the EO-triggered activation of the NKA signaling function (Rogachevskii et al., 2023). In comparison with the free ouabain molecule OUA, two additional hydrogen bonds between the rhamnosyl ring oxygens and NKA are identified in both EO5–NKA complexes, whereas less such hydrogen bonds are detected in the EO3–NKA complexes.

In addition to the steric accommodation, the chelated cation also has to be accommodated electrostatically, which means that its positive charge should be compensated for effective ligand–NKA binding. In other words, negatively charged Glu or Asp carboxylate anions of NKA should be accessible close to the cation. All ligands studied herein and OUA dock within the binding pocket at a similar depth which is determined rather strictly by the ligand–NKA hydrogen bond network. Hence, the cation position cannot be compromised much without its dissociation from the ouabain molecule after docking. On the other hand, it is difficult to expect a significant structural NKA rearrangement that would bring some distant anionic groups in close contact with the chelated cation.

Quite unexpectedly and fortuitously, the double positive charge of the chelated Ca^2+^ is completely compensated with two negatively charged Glu116 and Glu117 carboxylate anions upon EO5 binding to both NKA structures. Upon EO3 binding, on the contrary, the uncompensated Ca^2+^ charge results in the cleavage of the chelating bonds and in a loss of the ligand integrity, which indicates that EO3 is hardly a candidate for the role of endogenous ouabain. Therefore, the strong intermolecular ionic bonds formed by the chelated Ca^2+^ with Glu116 and Glu117 anions, and, in a lesser extent, two additional hydrogen bonds present in the EO5–NKA complexes are the molecular determinants which differentiate the EO binding from the OUA binding and which are thus responsible for the EO-triggered activation of NKA signaling. The data obtained indicate that the structure of EO5 molecule is finely tuned to very effectively bind to the OUA binding site in the NKA molecule. The EO5–NKA binding is demonstrated to be ∼1.5 kcal/mol more energetically favorable than the OUA–NKA binding. We suggest that this additional energy, together with possible NKA conformational changes correlated with the formation of intermolecular ionic bonds between the chelated Ca^2+^ and Glu116 and Glu117 NKA residues, mainly accounts for the EO5 ability to activate the NKA signaling function, as opposed to the modulation of the NKA pumping function by OUA.

Finally, a very solid argument indicating that EO is indeed the Ca^2+^ chelate complex of ouabain has been obtained *in vivo* using the formalin test. The strong antinociceptive effect at both the spinal and supraspinal levels has been observed only upon administration of the physiological solution containing ouabain preincubated with Ca^2+^, whereas the solution containing ouabain preincubated with Na^+^ failed to produce any statistically significant antinociceptive effect. This result correlates with the docking calculations which demonstrate that the model OUA-Na chelate complex binds to NKA less effectively than EO5: the single positive charge of the chelated Na^+^ does not suffice to electrostatically compensate for two Glu116 and Glu117 negative charges located in the cation’s vicinity, no additional ligand–NKA hydrogen bonds are formed as compared to the OUA binding, and the ligand–NKA binding is 0.8 kcal/mol less energetically favorable. Also quite surprisingly, OUA-Na has not been found to dock with α1R-NKA in the correct ligand orientation. Thus, according to the data obtained in the formalin test, it is only the administration of EO in a very low dosage and not of OUA-Na that evokes both the spinal and supraspinal antinociceptive effects at the organismal level. The subtle differences in the mechanisms of EO and OUA-Na binding with NKA elucidated by the docking studies account for the dramatic effect observed when CaCl_2_ as the adjuvant in the ouabain solution has been replaced with NaCl, the total loss of any analgesic activity upon administration of OUA-Na instead of EO.

It has to be noted that OUA cannot be used as the control substance in the formalin test, because its molecular form after the intraperitoneal injection is difficult to predict. Given the observed differences between the EO and OUA-Na effects, it is only EO that is demonstrated to be stable enough to survive the delivery to its molecular target as a chelate complex. The mechanisms for alleviation of acute (Ph1) and inflammatory pain (Ph2) are complicated (Butkevich et al., 2021). The manifestation of EO alleviating effect in the formalin test depends on the specific mechanisms of the first and second phases. But unfortunately, despite the success in studying the mechanisms of formalin-induced pain with the use of molecular genetic and pharmacological approaches, the extent to which the second phase depends both on the first phase and the interphase is not yet clear enough.

In our prior publication, it has been convincingly demonstrated that extremely low (endogenous) concentrations of EO interact with a new target in the nociceptive neuron, NKA, making it function as the signal transducer. As a result, two signaling cascades are activated: the tangential cascade along the neuron membrane to the Na_V_1.8 channel, and the downstream cascade to the SCN10A gene controlling the Na_V_1.8 channel expression (Plakhova et al., 2020). Additionally, EO-triggered activation of NKA signaling inhibits the DRG neurite growth. In the present work, the molecular mechanism of EO–NKA binding is for the first time described at the atomic level using the docking methodology. The docking results also provide an explanation for the fact that Strophanthin-G, the well-known antiarrhythmic agent, has never been reported to produce any antinociceptive effect. Strophanthin-G contains ouabain as the medicinal substance and NaOH as the adjuvant (not CaCl_2_), which indicates that the adjuvant can play a major role in the observed physiological and pharmacological effects of a medicinal substance.

There is no other data available in the literature regarding the third EO-triggered NKA signaling pathway, which has been discovered by us using the highly sensitive quantitative immunofluorescence methods and confocal microscopy. EO triggers the NKA/Src/PKA/p38 MAPK-mediated activation of NF-κB, which decreases the amount of the GAP-43 protein in the nociceptive neuron and leads to the inhibition of DRG neurite growth. This result is of major practical significance, as the nerve tissue growth is also inhibited, in addition to the strong analgesic effect based on the modulation of the Na_V_1.8 channels. It is known that cancer-related pain is linked to accelerating cancer progression and metastasis, and the sensory nerves that innervate primary tumors and metastases contribute to tumor-associated pain (Kuol et al., 2018). Therefore, suppression of the GAP43 functional activity might inhibit both cancer progression and metastasis, and EO is an excellent candidate for the role of both analgesic and cytostatic medicinal substance applied for the treatment of tumor-associated pain syndromes. Thus far, the possible EO cytostatic function remains a theoretical implication pending direct cancer-model validation.

One of the key advantages of our methodology, outlined in a series of recent publications (Penniyaynen et al., 2019; Plakhova et al., 2020; Plakhova et al., 2022; Rogachevskii et al., 2022; Rogachevskii et al., 2023; Kalinina et al., 2023; Plakhova et al., 2024), is the use of rats and chick embryos as warm-blooded animal models for investigating nociception mechanisms within a single study. It should be noted that experiments on rodents and large animals face ethical, practical, and technical challenges that limit their use. According to European regulations, interventions and procedures on chick embryos are not considered animal experiments. Moreover, they are even considered a replacement method in the context of the 3R principles (Aleksandrowicz and Herr, 2015). Chick embryos stand out as a unique research subject at the tissue and cellular levels (including downstream cascade studies) not only because of an exceptional sensitivity of their neurite growth to various substances but also due to the nearly complete homology of the NKA structure between rats and birds (Fambrough and Bayne, 1983; Takeyasu et al., 1987).

It has to be explicitly stated that so far there is no direct biochemical evidence obtained regarding the EO existence *in vivo* at physiological concentrations. EO formation and stability under physiological conditions remain hypothetical and require biochemical verification by spectrometric and chromatographic methods. However, this is a very technically challenging problem due to extremely low (nanomolar) concentrations of EO to be detected, which are below the sensitivity threshold of many experimental analytical techniques. Indirectly, the total absence of OUA-Na effect in the formalin test indicates a special role for the calcium cation in the manifestation of the EO analgesic effect at the organismal level.

## Data Availability

The original contributions presented in this study are included in the article/supplementary material, further inquiries can be directed to the corresponding authors.
